# Metabolomic and genetic associations with insulin resistance in pregnancy

**DOI:** 10.1007/s00125-020-05198-1

**Published:** 2020-06-18

**Authors:** Yu Liu, Alan Kuang, Octavious Talbot, James R. Bain, Michael J. Muehlbauer, M. Geoffrey Hayes, Olga R. Ilkayeva, Lynn P. Lowe, Boyd E. Metzger, Christopher B. Newgard, Denise M. Scholtens, William L. Lowe, HAPO Study Cooperative Research Group

**Affiliations:** 1 Department of Medicine, Northwestern University Feinberg School of Medicine, Rubloff 12, 420 E. Superior St, Chicago, IL 60611, USA; 2 Department of Endocrinology, South Campus, Renji Hospital, Shanghai JiaoTong University, Shanghai, China; 3 Department of Preventive Medicine, Northwestern University Feinberg School of Medicine, 680 N Lake Shore Drive, Suite 1400, Chicago, IL 60611, USA; 4 Sarah W. Stedman Nutrition and Metabolism Center, Duke University Medical Center, Durham, NC, USA; 5 Duke Molecular Physiology Institute, Durham, NC, USA; 6 Department of Medicine, Duke University School of Medicine, Durham, NC, USA

**Keywords:** *GCKR*, Genome-wide association studies, Insulin sensitivity, Metabolomics, Pregnancy

## Abstract

**Aims/hypothesis:**

Our study aimed to integrate maternal metabolic and genetic data related to insulin sensitivity during pregnancy to provide novel insights into mechanisms underlying pregnancy-induced insulin resistance.

**Methods:**

Fasting and 1 h serum samples were collected from women in the Hyperglycemia and Adverse Pregnancy Outcome Study who underwent an OGTT at ~28 weeks’ gestation. We obtained targeted and non-targeted metabolomics and genome-wide association data from 1600 and 4528 mothers, respectively, in four ancestry groups (Northern European, Afro-Caribbean, Mexican American and Thai); 1412 of the women had both metabolomics and genome-wide association data. Insulin sensitivity was calculated using a modified insulin sensitivity index that included fasting and 1 h glucose and C-peptide levels after a 75 g glucose load.

**Results:**

Per-metabolite and network analyses across the four ancestries identified numerous metabolites associated with maternal insulin sensitivity before and 1 h after a glucose load, ranging from amino acids and carbohydrates to fatty acids and lipids. Genome-wide association analyses identified 12 genetic variants in the glucokinase regulatory protein gene locus that were significantly associated with maternal insulin sensitivity, including a common functional missense mutation, rs1260326 (β=−0.2004, *p*=4.67×10^−12^ in a meta-analysis across the four ancestries). This SNP was also significantly associated with multiple fasting and 1 h metabolites during pregnancy, including fasting and 1 h triacylglycerols and 2-hydroxybutyrate and 1 h lactate, 2-ketoleucine/ketoisoleucine and palmitoleic acid. Mediation analysis suggested that 1 h palmitoleic acid contributes, in part, to the association of rs1260326 with maternal insulin sensitivity, explaining 13.7% (95% CI 4.0%, 23.3%) of the total effect.

**Conclusions/interpretation:**

The present study demonstrates commonalities between metabolites and genetic variants associated with insulin sensitivity in the gravid and non-gravid states and provides insights into mechanisms underlying pregnancy-induced insulin resistance.

## Graphical Abstract

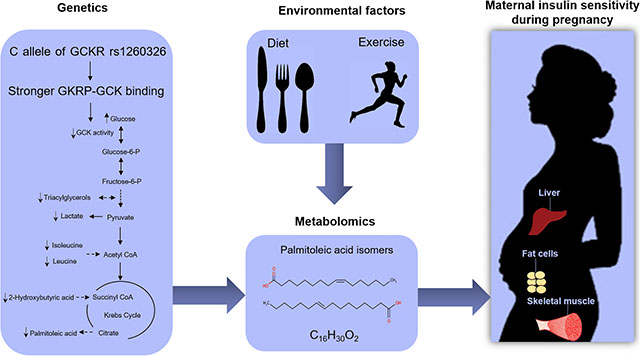

## Introduction

Human pregnancy is characterised by decreased insulin sensitivity with accompanying beta cell compensation to help meet maternal metabolic needs and ensure fetal growth and development. In the first trimester, maternal insulin sensitivity improves slightly but decreases by 30–70% in the second and third trimesters [1]. Secretion of adipokines (e.g. leptin) and cytokines (e.g. TNF-α, IL-6 and IL-1β), oxidative stress and, possibly, the gut microbiome contribute to pregnancy-induced insulin resistance [2], although understanding of pregnancy-induced insulin resistance is incomplete. Integration of multiple approaches, e.g. genetics and metabolomics, could help to identify underlying contributors to pregnancy-induced insulin resistance.

Dysregulation of interrelated pathways of glucose, lipid and amino acid metabolism contribute to insulin resistance in the non-gravid state. For example, human and animal studies have demonstrated a role for branched-chain amino acids (BCAAs) and their metabolic by-products as both a biomarker and causal agent of insulin resistance [3], while epidemiology studies have demonstrated accumulation of acylcarnitines and altered fatty acid and lipid metabolism in obesity-induced insulin resistance [4]. To date, metabolomics data related to pregnancy-induced insulin resistance are limited, and whether changes similar to those observed in non-pregnant cohorts are present is unclear.

Genome-wide association studies (GWAS) have identified genetic loci contributing to insulin resistance in non-gravid cohorts [5], but GWAS examining genetic variants associated with pregnancy-induced insulin resistance have not been reported. Again, whether similar variants are associated with insulin resistance in the gravid and non-gravid states is not known.

The goals of the present study were to characterise the maternal metabolome associated with insulin resistance during pregnancy before and after a glucose load, identify genetic loci associated with insulin resistance during pregnancy, and determine the interrelationship of associated genetic variants with the maternal metabolome.

## Methods

### Participants and data and sample collection

The Hyperglycemia and Adverse Pregnancy Outcome (HAPO) study was an observational, multinational, epidemiological study conducted from 1999 to 2006 that recruited 25,505 women to explore associations of maternal glucose levels with adverse pregnancy outcomes [6]. All pregnant women <32 weeks’ gestation at 15 field centres in nine countries were eligible for enrolment in the HAPO study, except when specific exclusion criteria were present: (1) age <18 years at the time of the first interview; (2) planning to deliver at another hospital; (3) date of last menstrual period uncertain and no ultrasound estimation from 6 to 24 weeks’ gestation; (4) unable to complete an OGTT by 32 weeks’ gestation; (5) multiple pregnancy; (6) became pregnant through assisted reproductive technology; (7) unblinded blood glucose testing and/or diagnosis of gestational diabetes mellitus (GDM) during the pregnancy prior to enrolment in HAPO; (8) previous diagnosis of diabetes requiring treatment with medication outside pregnancy; (9) receiving treatment with oral glucocorticoids, thiazide diuretics, β-blockers, ACE inhibitors, phenytoin or antiretroviral agents; (10) known to be HIV-positive or to have hepatitis B or C; (11) participation in the HAPO study during a previous pregnancy; (12) inability to converse in language(s) used in field centre forms without an interpreter. Maternal race/ethnicity was based on maternal self-report. The HAPO study protocol was approved by the institutional review board of each field centre, and all participants gave their informed consent.

Standardised phenotypic data were collected using a rigidly defined, common protocol across field centres. Rigorous training and certification procedures were established for research personnel to ensure data accuracy and reliability and consistency across field centres. Fasting, 1 h and 2 h glucose levels and fasting and 1 h C-peptide levels were measured in a central laboratory before and after a 75 g glucose load during an OGTT performed between 24 and 32 weeks’ gestation but as close to 28 weeks as possible [7]. The OGTT was performed in the morning after at least 3 days of usual diet and physical activity and fasting for 8–14 h. Participants consumed a standard 75 g glucose load within 5 min. Physical activity during the OGTT was limited to walking in the clinic. Maternal blood samples during the OGTT were processed by field centres within 60 min of collection, stored at −20°C or −80°C for 1–6 weeks, shipped on dry ice to the HAPO central laboratory and stored frozen at −80°C until used for the metabolomics assays. A total of 2189 women were unblinded because they had glucose values that exceeded predetermined levels during the OGTT or failed to complete the study (mainly because they delivered at a different hospital), resulting in a final cohort of 23,316 women.

Insulin sensitivity using OGTT glucose and C-peptide (ISOGTT C-pep) values was calculated according to the equation of Radaelli et al, with a numerator adjustment for scaling: IS_OGTT C-pep_=1000/√(fasting plasma glucose×fasting C-peptide×G×C), where G and C are the means of fasting and 1 h plasma glucose (mmol/l) and C-peptide (μg/l), respectively [8]. Maternal height, weight, systolic BP (SBP) and diastolic BP (DBP) were measured at the time of OGTT. BP was obtained using an Omron 711 electronic BP monitor (Omron Corporation, Japan) after the participant had been seated for ≥5 min. After obtaining the BP, the participant was instructed to remain seated and the BP reading was repeated 1–2 min later. The mean of the two BP measurements was used. Mean BP was calculated using the formula: mean BP=1/3×SBP+2/3×DBP. Duplicate height measurements were obtained at the time of the OGTT. If the measurements differed by ≥1 cm, a third was taken. Weight was measured using a beam balance scale prior to the OGTT. A duplicate measurement was obtained, and a third measurement was obtained if the two measures differed by more than 0.5 kg. The woman’s age, pre-pregnancy weight, gestational age at the time of the OGTT, family history of diabetes and hypertension, ethnic group and history of cigarette smoking and alcohol consumption were obtained from the participant questionnaire.

Targeted and non-targeted metabolomics data from 1600 HAPO mothers, including 400 women each from Afro-Caribbean, Northern European, Mexican American and Thai cohorts, were obtained on maternal serum samples collected at ~28 weeks’ gestation at the time of the HAPO study OGTT as described [9]. Mothers were sampled to span the range of maternal glucose and BMI. Genome-wide SNP data were obtained from 4528 HAPO mothers, including 1126 Afro-Caribbean, 1380 Northern European, 830 Mexican American and 1192 Thai mothers [10]. A total of 1412 women had both metabolomics and genome-wide SNP data available.

### Conventional clinical, targeted and non-targeted metabolite measurements

Conventional clinical and targeted metabolites present in maternal serum were measured as described [9, 11]. Specifically, conventional clinical metabolites were measured using standard enzymatic chemistries on a Beckman Coulter Unicell DxC 600 clinical analyser (Beckman Coulter, USA). These metabolites included triacylglycerols, NEFA, lactate, glycerol and 3-hydroxybutyrate (3-OHB).

Targeted panels of amino acids and acylcarnitines were analysed by flow injection, electrospray-ionisation tandem MS and quantified by isotope or pseudo-isotope dilution using a Waters TQ triple quadrupole mass spectrometer, equipped with an Acquity LC system and with data handling in the MassLynx 4.1 environment (Waters Corporation, USA). In total, 64 conventional and targeted metabolites were analysed.

Non-targeted assays were performed using GC-MS as described [11]. Briefly, serum samples were extracted with methanol that was spiked with a retention time-locking internal standard of perdeuterated myristic acid. After methanol extraction, drying and derivatisation by methoximation and trimethylsilylation, samples were run in daily batches of 11 matched pairs of fasting and 1 h OGTT sera on a 7890B GC/5977B MS (Agilent Technologies, USA). Each batch also included three injections of a quality control (QC) sample and a process blank. QC samples consisted of uniform pools generated from aliquots of all sera in the study and were injected at the beginning, middle and end of each batch. GC-MS peaks were deconvoluted with AMDIS freeware (http://www.amdis.net/) [12] and annotated using the Agilent Fiehn GC-MS Metabolomics RTL Library [13] with additions from the laboratory at Duke University School of Medicine. QC data were used for batch correction to account for sensitivity variation on a feature-specific basis, and batch correction was performed as described using the metabomxtr R package (version 1.22.0) [14, 15]. In total, 71 non-targeted metabolites that were not available in the conventional clinical and targeted metabolite assays were included in the final analyses. These were reported in relative terms as AMDIS-deconvoluted, integrated peak areas (amdis.net), which were then log_2_ transformed. Thus, a total of 135 metabolites (conventional clinical, targeted and non-targeted metabolites) were included in the final analyses.

### Genome-wide genotyping and imputation

The approach for genome-wide genotyping and QC has been described [10]. In brief, Afro-Caribbean and Mexican American DNA samples were genotyped using the Illumina HumanOmni1M-Duo v3B SNP array (Illumina, USA), Northern European samples using the Illumina Human610-Quad v1B SNP array at the Broad Institute (Cambridge, MA, USA), and Thai samples using the Illumina HumanOmni1-Quad v1–0B SNP array at the Center for Inherited Disease Research (Baltimore, MD, USA) following agreed-upon protocols of the Gene, Environment Association Studies Consortium [16]. For the final analyses, exclusion criteria for samples and SNPs included poorly performing samples, misspecified sex, chromosomal anomalies, unintended sample duplicates, sample relatedness, low call rate, high number of Mendelian errors, departures from Hardy–Weinberg equilibrium (<1×10^−4^), duplicate discordance, sex differences in heterozygosity, low minor allele frequencies (<0.01) and/or low imputation quality score (<0.75) [10].

Genotype imputation was performed on the Michigan Imputation Server using Minimac3 (version 2.0.1) [17] and the Consortium on Asthma among African-ancestry Populations in the Americas reference panel for Afro-Caribbean samples [18], the Haplotype Reference Consortium v.r1–1 reference panel for European ancestry samples [19] and the 1000 Genomes phase 3 v5 reference panel for Mexican American and Thai samples [20]. Consistent strand assignments between the reference dataset and the QC-cleaned and -filtered datasets were ensured using the strand-checking utility of Minimac3. Strand was corrected and/or SNPs were removed where strandedness could not be resolved. The HAPO mother’s genotype was then imputed to the above reference panels. We used a conservative allelic *r*^2^ threshold of 0.9 to remove unreliably imputed SNPs. A total of 6,168,240 SNPs were analysed in meta-analyses across the four ancestry groups.

### Data analysis

#### Per-metabolite analysis

Acylcarnitines and 3-OHB levels were log-transformed before analysis to satisfy normality. Distributions of the other targeted metabolites were sufficiently normal, as were non-targeted metabolites following log_2_ transformation. Outlying metabolite values, defined as ≥5 SDs from the mean, and data from three women with more than ten outlying metabolites were excluded from the statistical analysis.

For targeted metabolites, per-metabolite linear regression models were used to estimate associations between insulin sensitivity and metabolites within each ancestry group. Regression models included adjustment for maternal mean arterial pressure, age and gestational age at the OGTT, field centre, newborn sex, and sample storage time (model 1), model 1 + maternal BMI at OGTT (model 2), model 1 + parity (model 3) and model 3 + maternal BMI at OGTT (model 4). For analysis of non-targeted metabolites, a mixture model approach [15], which considered both the levels of metabolites with detectable values and frequency of samples with undetectable values, was used to estimate the association of non-targeted metabolites with insulin resistance. The mixture model relies on a joint statistical likelihood of the presence or absence of a metabolite in each sample using a logistic model and, if present, its continuous abundance using a normal distribution for the log_2_-transformed MS peak areas. The statistical likelihood is then maximised to calculate β estimates representing association with phenotypes. Bootstrap sampling was used to determine SEs of the β estimates. Separate analyses were conducted for fasting and 1 h metabolite levels and for the change in metabolite levels from fasting to 1 h.

Random effects meta-analysis of within-ancestry results [21] was used to examine associations of maternal metabolites with maternal insulin sensitivity, using the metafor R package, version 2.0.0 (www.metafor-project.org). Effect heterogeneity across ancestry groups was described using *I*^2^ statistics and formally tested using Cochran’s *Q* to test whether effects were heterogeneous.

Benjamini–Hochberg false discovery rate (FDR) correction was applied to metabolite analyses [22]. A *p* value <0.05 after FDR adjustment was used to indicate statistical significance.

#### Network analysis

Network analyses using the igraph R package (version 2.0.1) [23] were used to model metabolite correlations and their associations with insulin sensitivity as described [9]. For these analyses, separate partial correlation networks for metabolites associated with insulin resistance were constructed. Nodes represented metabolites, and edges demonstrated conditional dependence of metabolite pairs. Graphical lasso using the glasso R package, version 1.7 (http://CRAN.R-project.org/package=glasso), was applied to residuals from linear regression of metabolites on potential confounders to establish a sparse estimate of the inverse covariance matrix for all metabolites. The graphical lasso algorithm identifies pairs of metabolites that demonstrate partial correlation with each other after adjusting for all other metabolites in the network, and it uses a penalty variable to shrink very low partial correlations to zero. Thus, the strongest pairwise relationships are retained and edges between metabolite pairs are used to represent what is called ‘conditional dependence’ in the network [24]. Spinglass clusters represent communities of nodes that are more tightly connected to each other than to other nodes in the network [25]. Spinglass clustering was applied to the graphical lasso networks to assist with description of communities of related metabolites.

#### Association of SNPs with maternal insulin sensitivity

Association of SNPs with maternal insulin sensitivity was determined using linear regression under an additive model adjusting for field centre (for Northern European mothers), the first two principal components, gestational age (weeks), maternal age, BMI, height, mean arterial pressure, parity, smoking (yes/no) and alcohol intake status (yes/no) at the OGTT. SNPTEST, version 2.5, was used to estimate the β and SE for each regression model and assess the significance of the association between SNPs and maternal insulin sensitivity. SE and β were combined across the four cohorts using meta-analysis under a fixed-effects model, weighting each stratum by sample size. Statistical significance was considered to be *p*<5×10^−8^ in the fully adjusted model.

#### Association of SNPs with metabolites

Association of genetic variants with metabolite levels was analysed using linear regression under an additive model adjusting for field centre (for Northern European mothers), the first two principal components, gestational age (weeks), maternal age, BMI, height, mean arterial pressure and fasting plasma glucose at OGTT for fasting metabolites (1 h plasma glucose at OGTT for 1 h metabolites). SNPTEST, version 2.5, was used as described above. Since 135 metabolites were analysed, a Bonferroni-corrected *p* value <0.05 was considered statistically significant.

#### Mediation analysis

Structural equation modelling was used to evaluate mediation models using the lavaan, version 0.6–4 [26], R package in R-3.5.3. Simple mediation models were controlled for field centre (for Northern European mothers), first two principal components, gestational age (weeks), maternal age, BMI, height, mean arterial pressure, parity, smoking (yes/no) and alcohol intake status (yes/no) at OGTT, and fasting plasma glucose for fasting metabolites (1 h plasma glucose for 1 h metabolites). The aim of mediation analysis was to explore whether association of the SNP rs1260326, which is in *GCKR* and encodes glucokinase regulatory protein (GKRP), with maternal insulin sensitivity is mediated through specific metabolites. This was done by quantifying the direct and indirect relationships between the independent variable (rs1260326), mediator (specific metabolites) and dependent variable (maternal insulin sensitivity). A *p* value <0.05 was considered significant in these exploratory analyses.

## Results

### Study population

Maternal demographic data for the 4528 HAPO mothers included in the genome-wide SNP analyses [10] and 1600 HAPO mothers in the metabolomics analyses [9] were reported previously (electronic supplementary material [ESM] Tables 1 and 2, respectively).

### Targeted and non-targeted metabolites associated with insulin sensitivity

#### Per-metabolite analysis

**Fig. 1:**
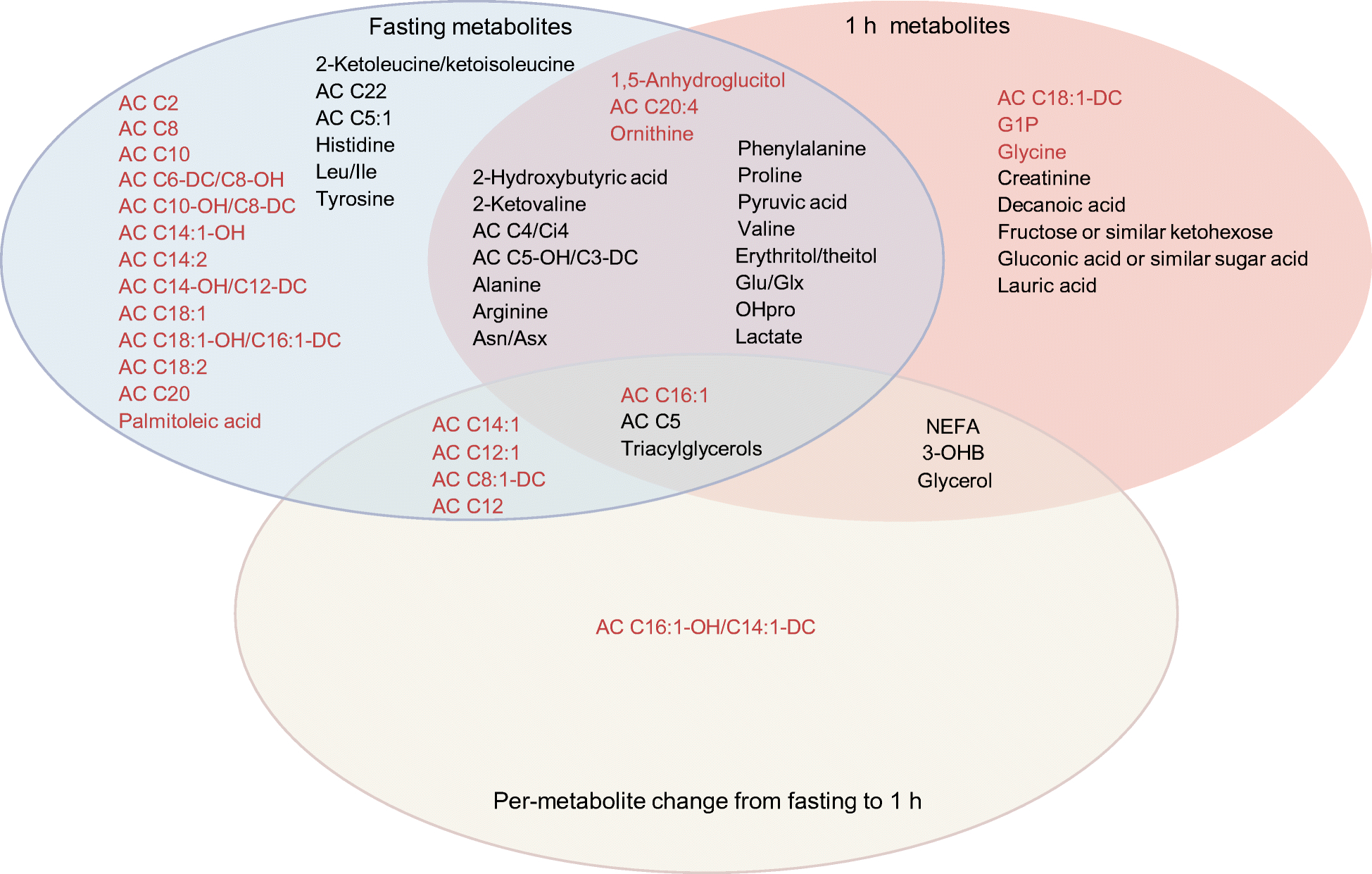
Significant associations of fasting and 1 h metabolite levels and per-metabolite change following a glucose load with insulin sensitivity in meta-analysis. Significant associations of metabolites are shown based on *p*<0.05 after FDR adjustment in the fully adjusted model (model 4), which included field centre, sample storage time, mean arterial pressure, maternal age, neonatal sex, gestational age and maternal BMI at OGTT and parity. The red metabolites were positively associated with insulin sensitivity and the black metabolites were inversely associated. AA, amino acid; AC, acylcarnitine; Asn/Asx, asparagine/aspartic acid; Cho, carbohydrate; FA, fatty acid; G1P, glycerol 1-phosphate; GC/TCA, glycolysis/tricarboxylic acid cycle; Glu/Glx, glutamine/glutamic acid; Leu/Ile, leucine/isoleucine; NM/2AA/NE, *N*-methylamine/2-aminobutanoic acid/*N*-ethylglycine; OA, organic acid; OHpro, hydroxyproline; Pur/Pyr, purine or pyrimidine

Initial analyses examined the association of maternal insulin sensitivity at ~28 weeks’ gestation with individual metabolite levels, both fasting and 1 h after a glucose load (Fig. 1; ESM Tables 3–5). Meta-analyses across the four ancestry groups demonstrated an inverse association of multiple fasting metabolites with maternal insulin sensitivity in model 4 (Fig. 1; ESM Table 4), including BCAAs, carnitine esters of their catabolites, BCAA-derived ketoacids and a BCAA-derived metabolic by-product, glutamate/glutamine, similar to what has been described in obese, insulin-resistant, non-pregnant adults [27, 28]. Additional amino acids inversely associated with insulin sensitivity included the aromatic amino acids tyrosine and phenylalanine as well as alanine, proline and its metabolite hydroxyproline, arginine, histidine and asparagine/aspartate. Other notable inversely associated metabolites included lactate, pyruvate, triacylglycerols, 2-hydroxybutyrate, docosanoyl carnitine (C22) and erythritol/threitol. Fasting metabolites positively associated with insulin sensitivity included multiple saturated and unsaturated short-, medium- and long-chain acylcarnitines as well as the non-proteinogenic amino acid ornithine, fatty acid palmitoleic acid and monosaccharide 1,5-anhydroglucitol.

Similar analyses identified metabolites associated with maternal insulin sensitivity 1 h after a glucose load (Fig. 1; ESM Table 5). With some exceptions, 1 h metabolites associated with insulin sensitivity were similar to those associated in the fasting state. This included inverse associations of valine, its ketoacid, carnitine esters derived from BCAA metabolism, glutamate/glutamine as well as the aromatic amino acid phenylalanine. Additional amino acids inversely associated with insulin sensitivity included proline and hydroxyproline, alanine, arginine and asparagine/aspartate. One hour glycine levels were positively associated with insulin sensitivity as were ornithine levels. Compared with the fasting state, a more limited number of long-chain, unsaturated acylcarnitines (C16:1, C18:1-DC, C20:4) were positively associated with insulin sensitivity. Similar to the fasting state, 1,5-anhydroglucitol was positively associated and erythritol/threitol inversely associated with insulin sensitivity. Additional sugars, including fructose and gluconate, were inversely associated with insulin sensitivity as were pyruvate, lactate and 2-hydroxybutyrate. Glycerol 1-phosphate was positively associated with insulin sensitivity, while triacylglycerols and additional lipid-related metabolites, including NEFA, the fatty acids decanoate and laurate, 3-OHB and glycerol, were inversely associated with insulin sensitivity. These latter features are consistent with a model in which more insulin-resistant individuals are less able to suppress peripheral lipolysis during a glucose challenge, resulting in higher levels of products of lipid mobilisation such as NEFA, ketones and glycerol. This may in turn influence glucose homeostasis by altering hepatic acetyl-CoA levels to promote gluconeogenesis [29].

The change in levels of a limited number of metabolites from fasting to 1 h following a glucose load was associated with maternal insulin sensitivity (ESM Fig. 1, ESM Table 6). This included an inverse association of the change in level of lipid-related metabolites, including triacylglycerols, glycerol, NEFA and 3-OHB, as well as the BCAA metabolite acylcarnitine C5. The change in level of a number of medium- and long-chain acylcarnitines was positively associated with insulin sensitivity.

Power was limited to examine fasting and 1 h metabolites associated with insulin sensitivity within each ancestry group. However, these exploratory analyses did demonstrate differences in the metabolites that reached statistical significance in their association with insulin sensitivity within the different ancestry groups (ESM Fig. 2, ESM Tables 4–6). Prominent among these were differences in the acylcarnitines that reached statistical significance in the different ancestry groups.

#### Network analysis

**Fig. 2:**
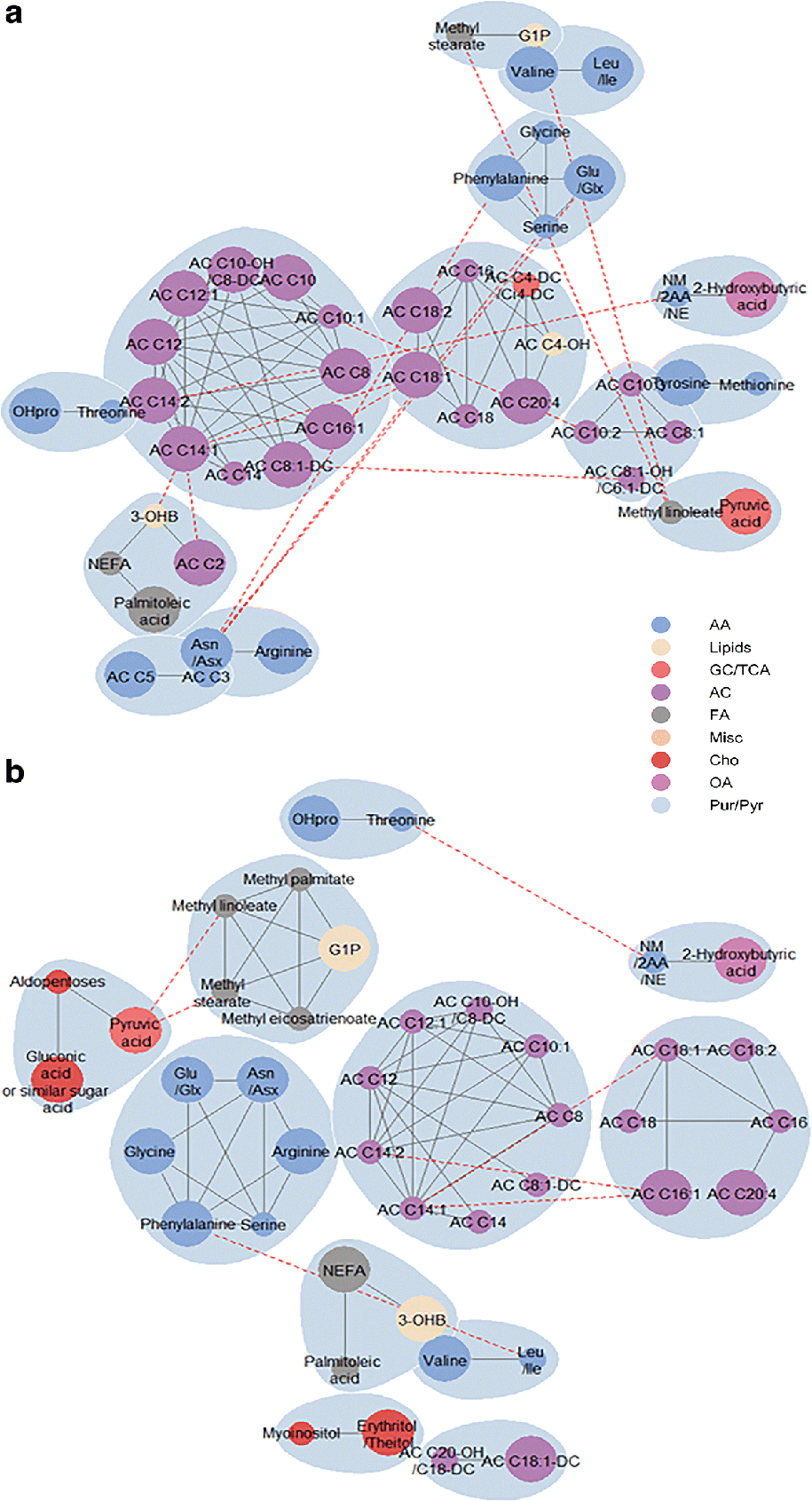
Sub-networks of fasting metabolites (**a**) and 1 h metabolites (**b**) associated with maternal insulin sensitivity. Nodes are coloured by metabolite class and sized by FDR-adjusted *p* values in the fully adjusted model (model 4), with large nodes referring to metabolites significantly associated with insulin sensitivity and small nodes referring to metabolites correlated with an individually significant metabolite. Blue shading represents spinglass communities within the estimated network. The solid lines between two nodes represent dependencies for intra-cluster metabolites, and the red dashed lines represent dependencies for metabolites across clusters. AA, amino acid; AC, acylcarnitine; Asn/Asx, asparagine/aspartic acid; Cho, carbohydrate; FA, fatty acid; G1P, glycerol 1-phosphate; Glu/Glx, glutamine/glutamic acid; Leu/Ile, leucine/isoleucine; NM/2AA/NE, *N*-methylamine/2-aminobutanoic acid/*N*-ethylglycine; OA, organic acid; OHpro, hydroxyproline; Pur/Pyr, purine or pyrimidine

Recognising that dependencies exist among metabolites, network analyses were conducted to better characterise joint associations of maternal insulin sensitivity with fasting and 1 h metabolites (Fig. 2). These analyses allow for visualisation of individual significant metabolite–phenotype associations in the context of a larger metabolite environment. Spinglass clusters, representing communities of metabolites more tightly connected to each other than to other metabolites in the network, were also identified [25].

The network of fasting metabolites associated with maternal insulin sensitivity in the fully adjusted model included 14 spinglass clusters, most of which were composed of acylcarnitines or amino acids. By contrast, the network of 1 h metabolites included a more diverse group of spinglass clusters with clusters of acylcarnitines and amino acids, together with clusters of lipid-related metabolites and carbohydrates.

### SNPs associated with insulin sensitivity and metabolites during pregnancy

**Fig. 3:**
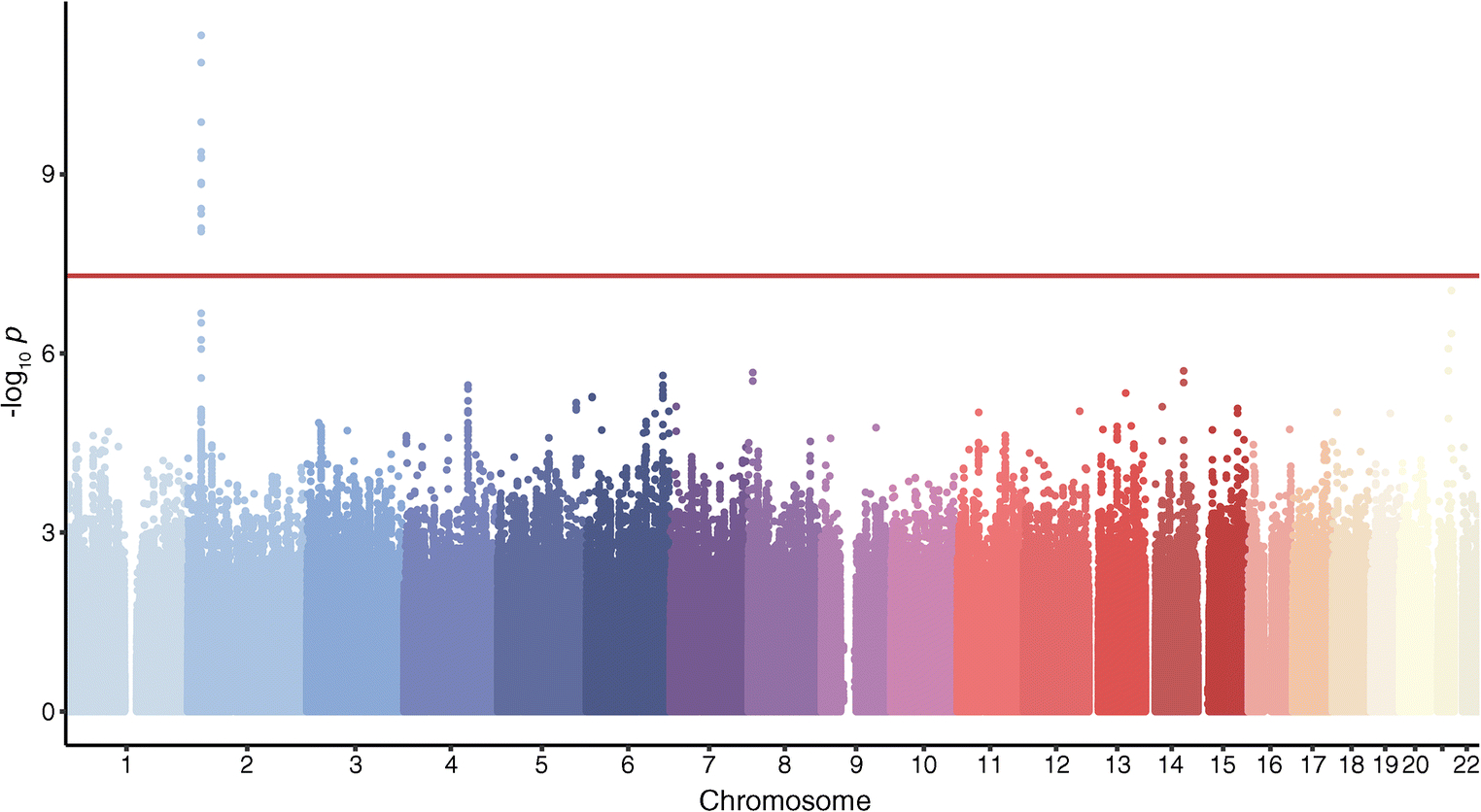
Manhattan plot for maternal insulin sensitivity for the meta-analysis across the four ancestry groups. The red line indicates genome-wide significance (*p*<5×10^−8^)

We performed a GWAS using genome-wide SNP data from 4528 HAPO mothers in four ancestry groups to identify genetic variants associated with maternal insulin sensitivity at ~28 weeks’ gestation. In a meta-analysis across the four ancestry groups, 12 variants in the *GCKR* locus which were in linkage disequilibrium demonstrated a genome-wide significant association (*p*<5×10^−8^) with maternal insulin sensitivity (Fig. 3; ESM Tables 7, 8). These variants included rs1260326, which encodes a functional missense mutation (P446L). With proline (encoded by the C allele of rs1260326) as opposed to leucine (encoded by the T allele of rs1260326) at position 446, GKRP responds more robustly to fructose-6-phosphate, resulting in more avid binding of glucokinase to GKRP (with a resulting decrease in glucokinase activity) [30, 31].

As rs1260326 is thought to be the functional SNP that accounts for association of the pleiotropic *GCKR* locus with multiple metabolic phenotypes [31], its association with the levels of fasting and 1 h metabolites associated with insulin sensitivity was examined (Table 1; ESM Table 9). In a meta-analysis across the four ancestries, the C allele of rs1260326 demonstrated a significantly inverse association with fasting and 1 h triacylglycerols and 2-hydroxybutyrate as well as 1 h lactate, 2-ketoleucine/ketoisoleucine and palmitoleic acid. The proportion of variation in metabolite levels explained by rs1260326 varied from 4.1% in Northern Europeans to 1.5% in Thais for fasting 2-hydroxybutyrate, from 4.5% in Northern Europeans to 2.6% in Mexican Americans for 1 h 2-hydroxybutyrate, from 5.8% in Northern Europeans to 0.5% in Thais for fasting triacylglycerols, from 4.8% in Northern Europeans to 0.4% in Thais for 1 h triacylglycerols, from 3.2% in Northern Europeans to 0.7% in Afro-Caribbeans for 1 h 2-ketoleucine/ketoisoleucine, from 4.1% in Mexican Americans to 1.4% in Thais for 1 h lactate, and from 2.3% in Northern Europeans to 0.3% in Thais for 1 h palmitoleic acid.

### Mediation analysis

**Fig. 4:**
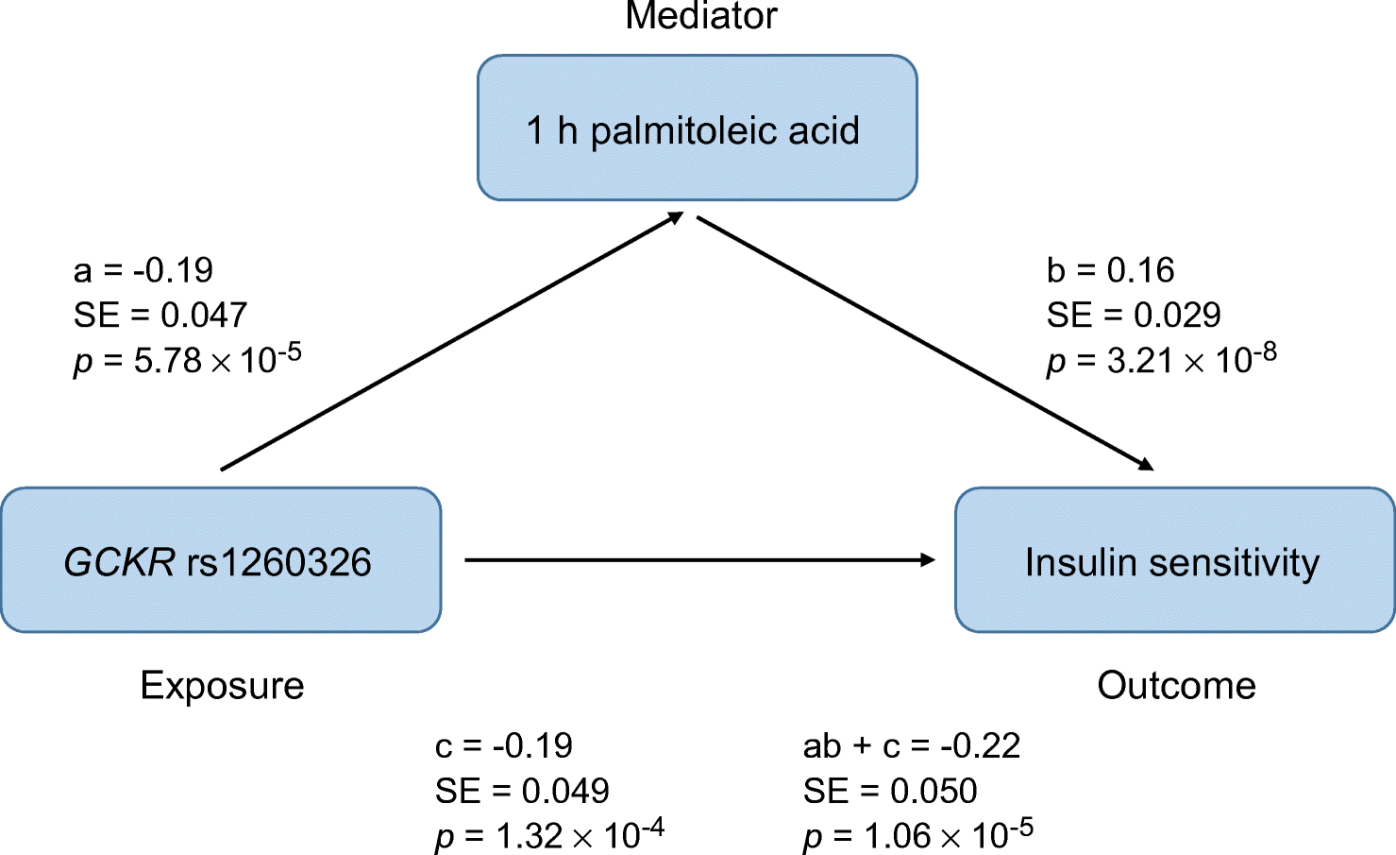
Mediation analysis of the role of 1 h palmitoleic acid in mediating the relationship between rs1260326 and maternal insulin sensitivity. a, The association between rs1260326 and 1 h palmitoleic acid; b, the association between 1 h palmitoleic acid and maternal insulin sensitivity; c, the direct association between rs1260326 and maternal insulin sensitivity after adjustment for 1 h palmitoleic acid; ab+c, the total effect of rs1260326 on maternal insulin sensitivity. All associations were adjusted for field centre (for European ancestry mothers), the first two principal components, gestational age (weeks), maternal age, BMI, height, mean arterial pressure, parity, smoking (yes/no), alcohol intake status (yes/no) and fasting plasma glucose for fasting metabolites (1 h plasma glucose for 1 h metabolites) at the OGTT

We next tested whether the metabolites associated with both maternal insulin sensitivity and rs1260326 (fasting and 1 h triacylglycerols and 2-hydroxybutyrate; 1 h lactate, 2-ketoleucine/ketoisoleucine and palmitoleic acid) mediated, in part, the relationship between rs1260326 and maternal insulin sensitivity (Fig. 4; ESM Table 10). Given the cross-sectional nature of the data, these analyses reflected hypothesised directionality of associations.

In structural equation models, the association of rs1260326 with 1 h palmitoleic acid was statistically significant (β=−0.19, SE=0.047, *p*=5.78×10^−5^), as was the association of 1 h palmitoleic acid with maternal insulin sensitivity (β=0.16, SE=0.029, *p*=3.21×10^−8^). The indirect effect of rs1260326 on maternal insulin sensitivity through 1 h palmitoleic acid was also statistically significant (β=−0.030, SE=0.0092, *p*=1.14×10^−3^), as was the direct effect of rs1260326 on maternal insulin sensitivity with 1 h palmitoleic acid modelled as the hypothesised mediator (β=−0.19, SE=0.049, *p*=1.32×10^−4^). The total direct effect for rs1260326 on maternal insulin sensitivity yielded regression coefficients of greater magnitude than the model that included the hypothesised mediator 1 h palmitoleic acid (β=−0.22, SE=0.050, *p*=1.06×10^−5^). These results suggest that 1 h palmitoleic acid mediates, in part, the relationship between rs1260326 and maternal insulin sensitivity, accounting for 13.7% (95% CI 4.0%, 23.3%) of the total effect size for rs1260326.

For some metabolites, the indirect effects in structural equation models reached significance for rs1260326 (fasting 2-hydroxybutyrate, fasting and 1 h lactate and triacylglycerols). However, the direct effect of rs1260326 on maternal insulin sensitivity was larger than the total effect and had an opposite direction compared with the indirect effect. This suggests that these metabolites had a suppressive effect on the relationship between rs1260326 and maternal insulin sensitivity.

## Discussion

Metabolomic profiling to better define pregnancy-induced insulin resistance has received limited attention. Previously, we reported the association of targeted and conventional clinical metabolites with maternal insulin sensitivity at ~28 weeks’ gestation [32]. We have now expanded on those results through inclusion of non-targeted metabolites and analyses of metabolic networks. Together, the targeted assays of acylcarnitines, amino acids and conventional clinical metabolites, and non-targeted assays, cover the small molecules in the major human bioenergetics pathways, including glycolysis, the Krebs cycle, ketones, β- and ω-oxidation of fatty acids, and catabolism of amino acids. The non-targeted metabolites also provide some insight into metabolism of diverse small sugars, sugar alcohols, sugar acids, purines, pyrimidines and certain botanical foodstuffs and gut-microbial metabolites that enter the human circulation. Integration of genetic and metabolomics data in the present study demonstrated that one metabolite associated with maternal insulin sensitivity, palmitoleic acid, mediates, in part, the association of a genetic variant with insulin resistance.

We observed an association of higher levels of BCAAs, their branched-chain ketoacids and metabolic by-products with maternal insulin resistance, similar to that of the non-gravid state [27, 28]. In the non-gravid state, it has been hypothesised that BCAA accumulation contributes to insulin resistance by inducing incomplete oxidation of fatty acids in skeletal muscle [33]. Consistent with that, Zucker obese rats fed a standard chow/BCAA-restricted diet shifted from glucose to fatty acid oxidation in the heart compared with Zucker obese rats maintained on a standard chow diet [34]. Whether a similar mechanism holds in pregnancy is not known. In non-gravid cohorts, aromatic amino acids were also associated with insulin resistance [27]. Previously, phenylalanine, but not tyrosine, was shown to be associated with fasting C-peptide during pregnancy [35], consistent with our finding that phenylalanine was associated with maternal insulin resistance. In our study, tyrosine, the initial product of phenylalanine catabolism, was also associated with insulin resistance. In contrast to a previous study [36], we also demonstrated an association of histidine, a precursor of glutamate and α-ketoglutarate, with maternal insulin resistance.

The extent to which the mechanisms underlying insulin resistance in pregnancy differ from those of the non-gravid state is not known [37]. Numerous metabolites are associated with insulin resistance in both gravid and non-gravid cohorts; however, one notable difference is the acylcarnitines. Accumulation of acylcarnitines, which can either reflect mitochondrial dysfunction or increased rates of fatty acid oxidation, is associated with insulin resistance in non-gravid cohorts: for example, medium-chain but not short- and long-chain acylcarnitines were associated with insulin resistance in non-pregnant populations [38, 39]. Here we found that short-, medium- and long-chain acylcarnitines were positively associated with maternal insulin sensitivity during pregnancy, suggesting differences in the association of acylcarnitines with insulin sensitivity in the pregnant vs the non-pregnant state. Better understanding of the mechanistic significance of this finding will require examination of acylcarnitine levels in tissue biopsies. For example, in skeletal muscle of rodent models of obesity, increases in a broad array of acylcarnitines were accompanied by decreased tricarboxylic acid cycle intermediates, indicative of impaired mitochondrial fatty acid metabolism [4]. This lesion may not be present in maternal skeletal muscle during pregnancy.

Candidate gene and GWAS have identified genetic variants associated with insulin resistance in the non-gravid state, including variants in *GCKR* [5, 40]. Here we performed a GWAS to identify SNPs associated with insulin sensitivity in pregnancy, which, to our knowledge, has not been previously reported. Multiple SNPs within *GCKR* reached genome-wide significance. The modest size of the cohort likely limited power; thus, additional loci may await identification in larger cohorts. Variants in *GCKR*, including rs1260326, have been associated with higher glucose, insulin resistance and type 2 diabetes in non-gravid cohorts [31, 41]. In our study, the C allele of rs1260326 was associated with greater insulin resistance.

Multiple studies have demonstrated that the *GCKR* locus is a pleiotropic locus associated with a wide variety of metabolites and metabolic phenotypes, with the strongest association found with triacylglycerol levels [31]. However, the interface between *GCKR* variants in the *GCKR* locus, altered glucose metabolism and metabolite levels in the non-gravid state is somewhat paradoxical in that the C allele of rs1260326 is associated with a greater risk of type 2 diabetes, higher levels of fasting glucose and insulin as well as insulin resistance, but lower levels of fasting triacylglycerols, alanine, leucine and isoleucine [42–44]. The association of rs1260326 with metabolite levels in pregnancy has not been examined previously, either in the fasting state or following a glucose load. We have now demonstrated an inverse association of the C allele of rs1260326 with multiple metabolites during pregnancy, including fasting and 1 h triacylglycerols and 2-hydroxybutyrate, as well as 1 h lactate, palmitoleic acid and 2-ketoleucine/isoleucine, a BCAA metabolite. An inverse association of rs1260326 with 2-hydroxybutyrate has been reported in non-gravid populations; 2-hydroxybutyrate is associated with insulin resistance [45] and was reduced after gastric bypass, as weight and insulin resistance decreased [46]. The inverse association of rs1260326 with 1 h lactate levels and fasting and 1 h triacylglycerol levels is consistent with its inverse association with fasting lactate and triacylglycerols in non-gravid cohorts [43, 47]. These findings with triacylglycerols and 2-hydroxybutyrate levels are somewhat paradoxical given the positive association of the C allele of rs1260326 with insulin resistance and its inverse association with the levels of these metabolites in both non-gravid [31] and, as shown here, gravid cohorts. The mechanisms underlying these paradoxical associations remain to be determined, although the C allele of rs1260326 is likely associated with lower hepatic glucokinase activity [31], which would result in less effective glucose clearance, lower rates of de novo lipogenesis and a state of ‘insulin resistance’.

The availability of both genetic and metabolomic data related to maternal insulin sensitivity during pregnancy allowed use of mediation analyses to define the potential role of metabolites in pregnancy-induced insulin resistance. Serum palmitoleic acid levels were inversely associated with insulin sensitivity in our cohort, while the C allele of rs1260326 was associated with lower insulin sensitivity, and carriers of this allele had lower levels of palmitoleic acid. Mediation analyses indicated that 1 h palmitoleic acid mediated, in part, the association of rs1260326 with maternal insulin resistance. Palmitoleic acid is the second most abundant monounsaturated fatty acid in the circulation and is derived primarily from endogenous synthesis in adipose tissue and to some extent dietary intake [48, 49]. Women with GDM have lower levels of palmitoleic acid [50], while some, but not all, studies in non-gravid cohorts have demonstrated a positive association of circulating levels of palmitoleic acid with insulin sensitivity [51]. More recently, a longitudinal study in humans demonstrated that palmitoleic acid was an independent determinant of changes in insulin sensitivity [48]. Finally, in rodents, infusion of palmitoleic acid improved whole body glucose disposal and hepatic insulin sensitivity [52, 53]. Together, and consistent with the mediation analyses, these studies support a potential role for palmitoleic acid as an adipocyte-derived lipokine that enhances insulin sensitivity [52, 54].

This study had several strengths: it included a large cohort of pregnant women from multiple ancestries, ensuring applicability of the results to diverse populations; the availability of samples from the fasting state and 1 h after a glucose load allowed insight into two different metabolic states; and the genetic data allowed for extension of the results beyond associations. The study also had limitations: first, the study was cross-sectional, limiting the ability to establish causality; second, the gold standard for measuring insulin sensitivity is the hyperinsulinaemic–euglycaemic clamp, but this approach was not practical for a large population-based study, and the equation used to estimate insulin sensitivity was validated against this technique [55]; third, as the current study captured only a portion of the metabolome that is detectable using a GC-MS-based technology, additional studies will be needed to explore the potential contribution of additional metabolites; finally, the GWAS findings were not replicated. Similar associations of rs1260326 with the phenotypes and metabolites demonstrated here have, however, been reported in non-gravid cohorts.

In conclusion, this is the first report of integrating genetic and metabolomic data to better characterise maternal metabolism during pregnancy. Numerous metabolites, including amino acids, carbohydrates, fatty acids and lipids, were associated with maternal insulin sensitivity, independently of maternal BMI, while common variants in *GCKR* were significantly associated with multiple fasting and 1 h metabolites during pregnancy. Among the significant metabolites, mediation analyses suggested that 1 h palmitoleic acid might underlie, in part, the association of rs1260326 with maternal insulin sensitivity. These studies have begun to define mechanisms underlying pregnancy-induced insulin resistance. Future studies in larger cohorts will provide the opportunity to better define these mechanisms.

## Supplementary Material

125_2020_5198_MOESM1_ESM

125_2020_5198_MOESM2_ESM

## Figures and Tables

**Table 1:** Association of rs1260326 with metabolite levels

	β	SE	*p* value	*Q* value	*Q* test *p* value	*I*^*2*^
Fasting 2-hydroxybutyrate	−0.1492	0.0252	3.15×10^−9^	1.2323	0.7453	0
Fasting triacylglycerols	−13.8873	3.0867	6.82×10^−6^	4.4725	0.2148	31.3158
1 h 2-Hydroxybutyrate	−0.1703	0.0238	8.57×10^−13^	2.3580	0.5015	0
1 h 2-Ketoleucine/ketoisoleucine	−0.1616	0.0415	9.94×10^−5^	1.3029	0.7284	0
1 h Lactate	−0.1325	0.0230	7.96×10^−9^	3.3787	0.3368	16.1422
1 h Palmitoleic acid	−0.1765	0.0466	1.51×10^−4^	2.7775	0.4272	0
1 h Triacylglycerols	−13.7714	2.8257	1.10×10^−6^	3.6430	0.3027	15.8069

C allele is the effect allele for rs1260326

*p*<0.05 after Bonferroni correction and adjustment for field centre (for European ancestry mothers), the first two principal components, gestational age (weeks), maternal age, BMI, height, mean arterial pressure and fasting plasma glucose at OGTT for fasting metabolites (1 h plasma glucose at OGTT for 1 h metabolites) was considered to be statistically significant. See also ESM Table 7.

## Reserch in context

### What is already known about this subject?

- Pregnancy is associated with a marked increase in insulin resistance
- Secretion of a number of hormones and cytokines by the placenta and other tissues contributes to pregnancy-induced insulin resistance
- Changes in the maternal metabolome associated with maternal insulin resistance during pregnancy have not been clearly defined

### What is the key question?

- Are contributions of the maternal metabolome and genome to pregnancy-induced insulin resistance similar to those in the non-gravid state?

### What are the new findings?

- Our study identified numerous metabolites associated with maternal insulin sensitivity before and 1 h after a glucose load, ranging from amino acids and carbohydrate metabolites to fatty acids, other lipids and lipid metabolites
- Genome-wide association analyses identified 12 genetic variants in the *GCKR* locus significantly associated with maternal insulin sensitivity
- rsl 260326, a common functional missense mutation in *GCKR*, was significantly associated with multiple fasting and 1 h metabolites during pregnancy, including fasting and 1 h triacylglycerol and 2-hydro)cybutyrate_/_ and 1 h lactate, 2-ketoleucine/ketoisoleucine and palmitoleic acid, with mediation analyses suggesting that 1 h palmitoleic acid contributes, in part, to the association of rs 1260326 with pregnancy-induced insulin resistance

### How might this impact on clinical practice in the foreseeable future?

- Identification of metabolic and genetic markers associated with pregnancy-induced insulin resistance might inform new approaches for identifying women at risk of developing a greater degree of insulin resistance during pregnancy, and of gestational diabetes

## Abbreviations

BCAA: Branched-chain amino acid
FDR: False discovery rate
GDM: Gestational diabetes mellitus
GKRP: Glucokinase regulatory protein
GWAS: Genome-wide association studies
HAPO: Hyperglycemia and Adverse Pregnancy Outcome
3-OHB: 3-Hydroxybutyrate
QC: Quality control
