# Supplementary material for: Metabolomic and genetic associations with insulin resistance in pregnancy
Source: Diabetologia. Author manuscript; Available in PMC 2021 Sep 1. (PMC7416451; doi:10.1007/s00125-020-05198-1)
Supplement: 125_2020_5198_MOESM1_ESM [file NIHMS1605376-supplement-125_2020_5198_MOESM1_ESM.pdf]

ESM Figure 1

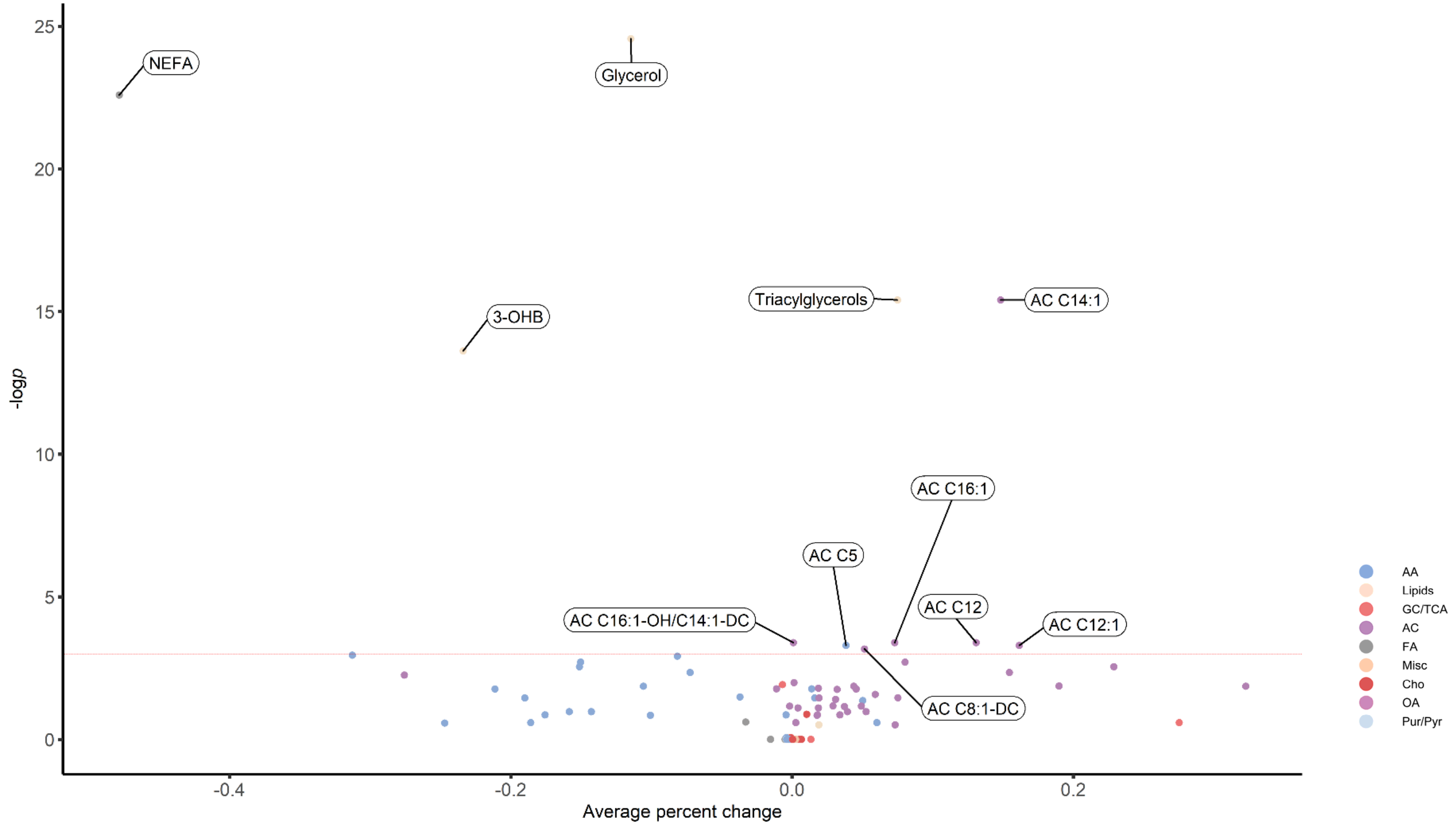

Volcano plot showing an association of per-metabolite change in levels from fasting to 1hr during an OGTT with insulin sensitivity. The y-axis shows the negative $\log_{10}$ transformation of FDR- adjusted *p*-values from fully adjusted linear regression models

evaluating associations of the percentage change in metabolite levels following a glucose load with insulin sensitivity in a meta-analysis across the four cohorts. The x-axis shows the average percent change in the level of metabolites from fasting to 1-h across the four ancestry groups. Points are colored indicating different metabolite groups. 3-OHB, 3-hydroxybutyrate; AA, amino acid; AC, acylcarnitine; Cho, carbohydrate; FA, fatty acid; GC/TCA, glycolysis/tricarboxylic acid cycle; Misc, miscellaneous; NEFA, non-esterified fatty acid; OA, organic acid; Pur/Pyr, purine or pyrimidine.

ESM Figure 2. a

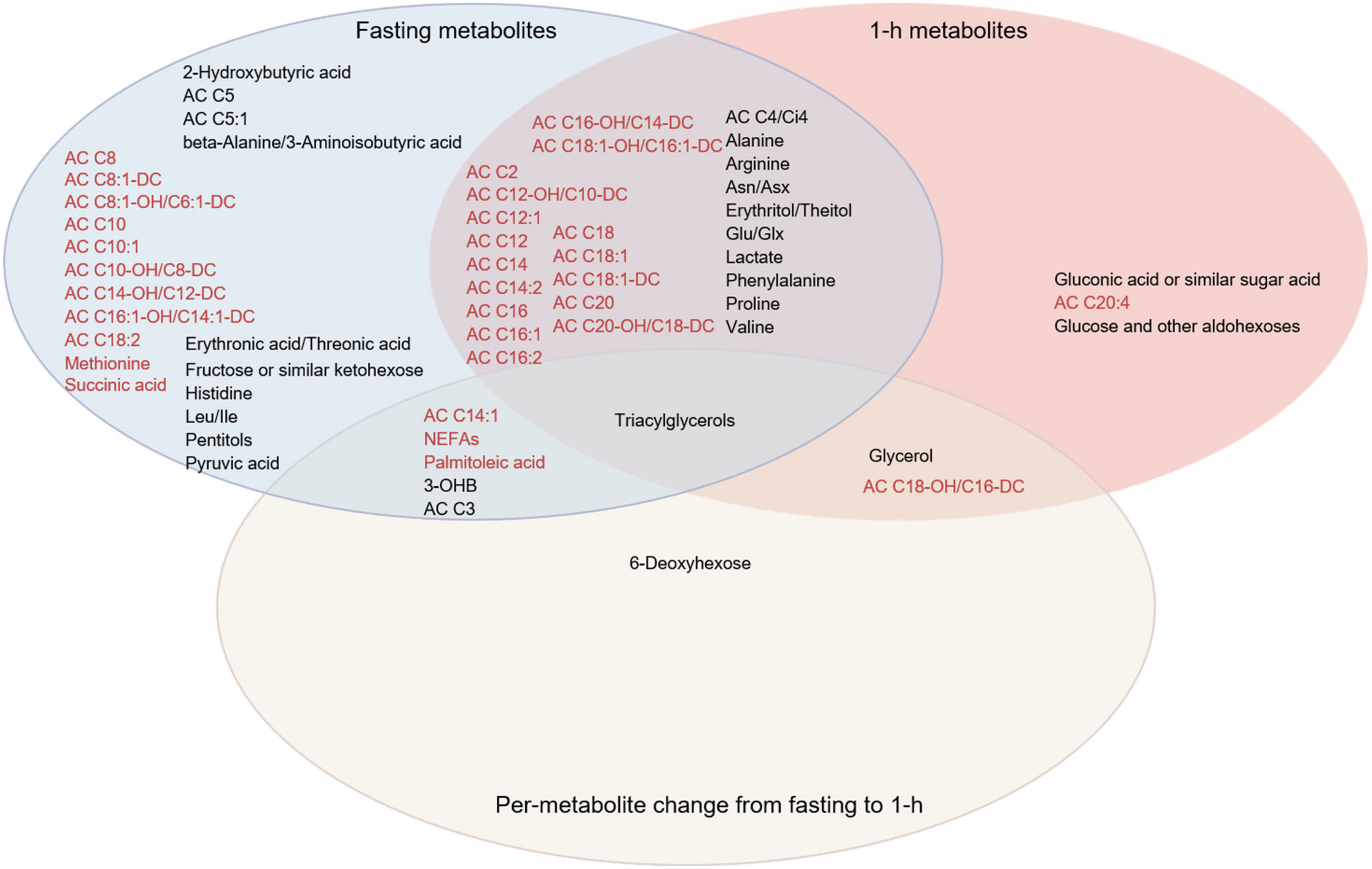

ESM Figure 2. b

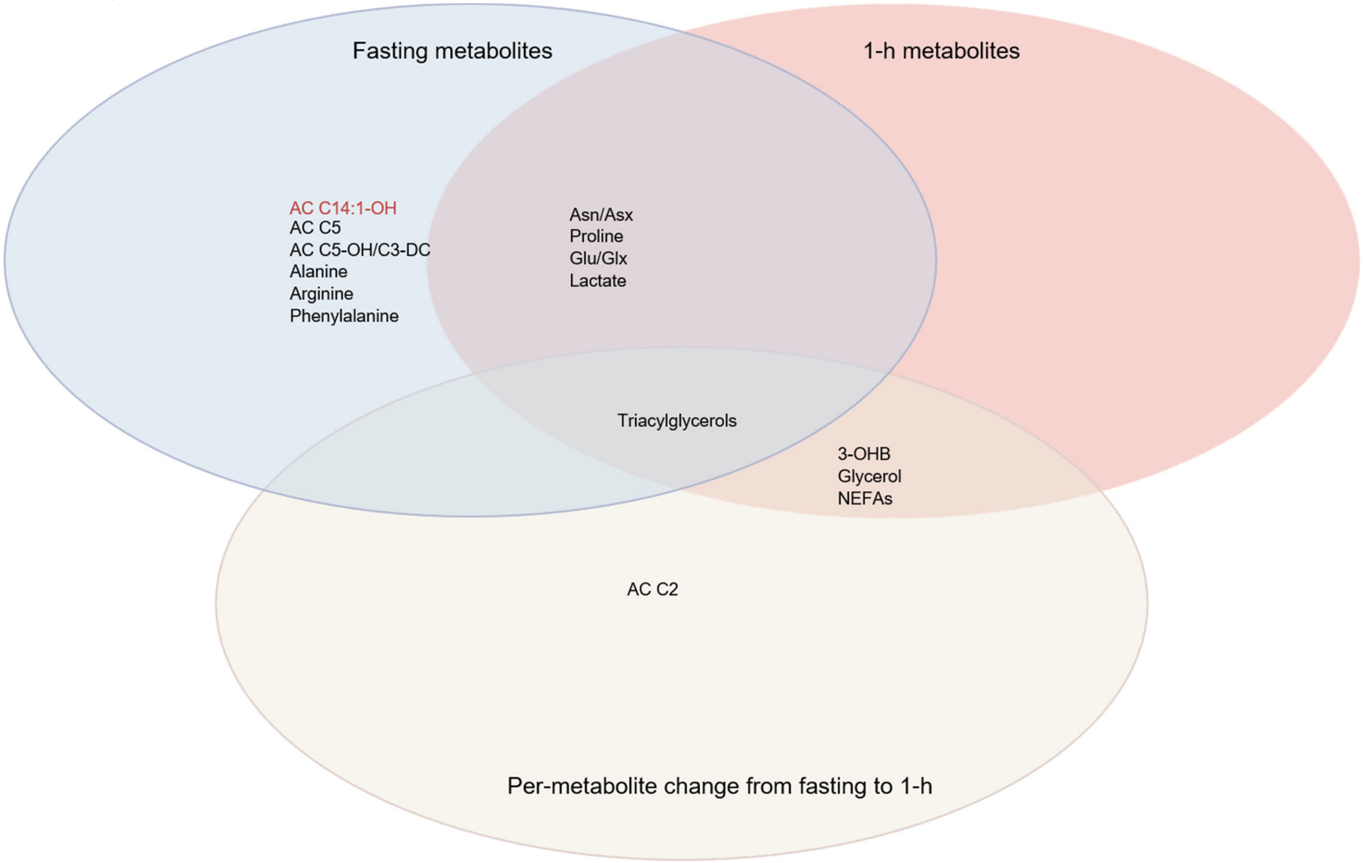

ESM Figure 2. c

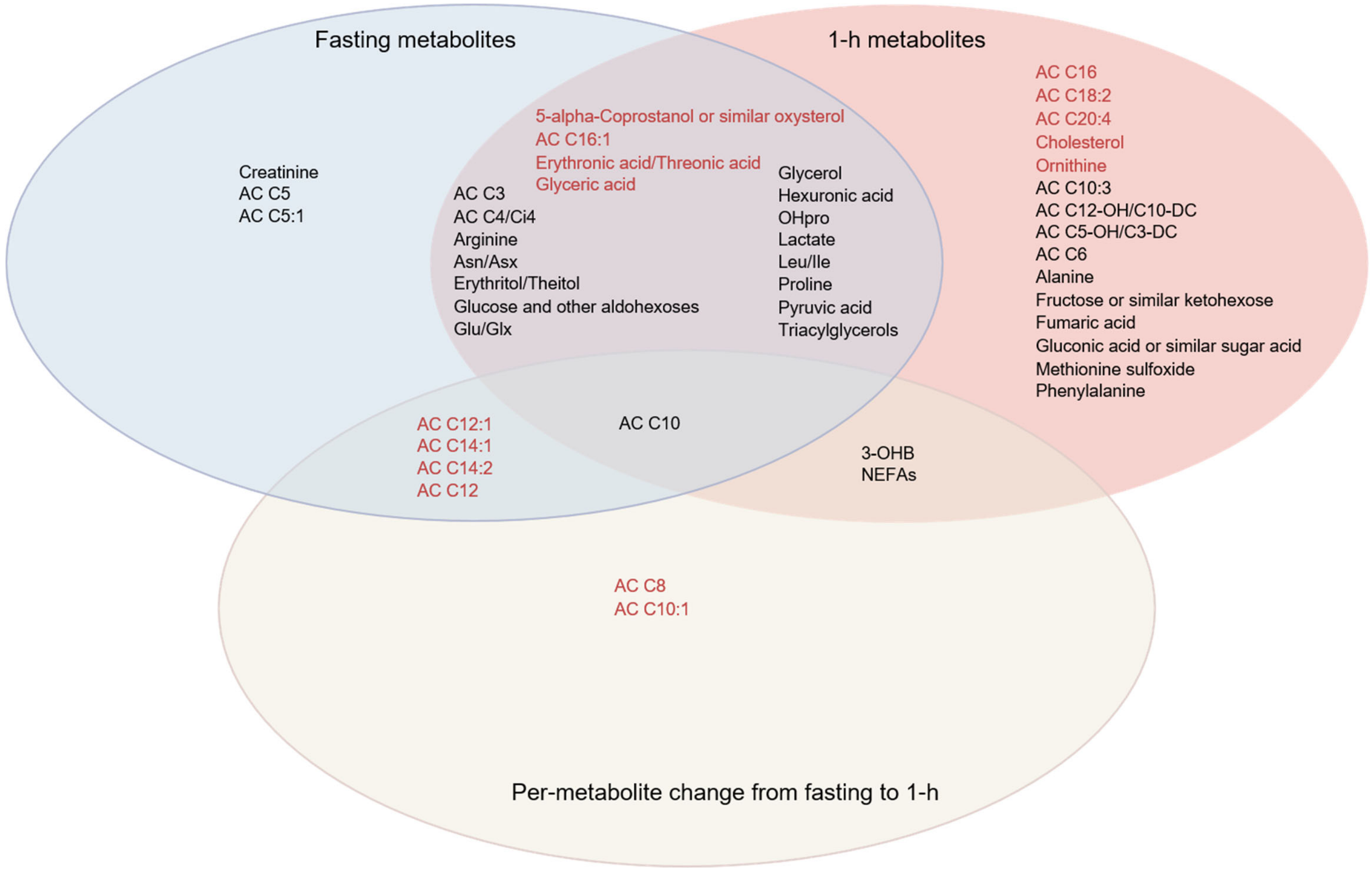

ESM Figure 2. d

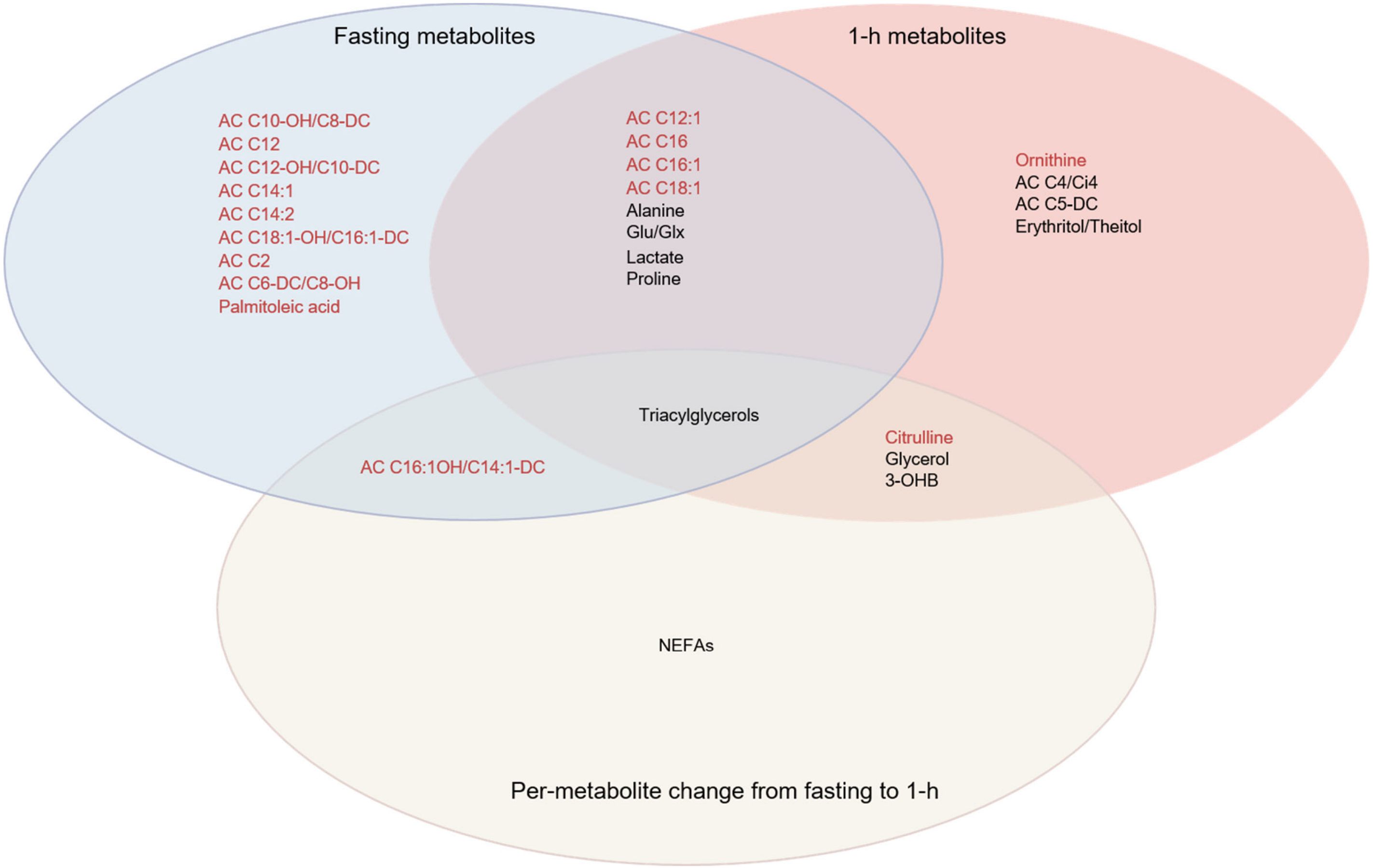

Significant associations of fasting and 1-h metabolite levels and per-metabolite change following a glucose load with insulin

sensitivity across the four ancestry groups. a: Afro-Caribbean, b: Northern European , c: Mexican American, d: Thai. Significant associations of metabolites are shown based on $p < 0.05$ after FDR adjustment in the fully adjusted model (Model 4), which included field center, sample storage time, mean arterial pressure, maternal age, neonatal sex, gestational age and maternal BMI at OGTT and parity. The red metabolites were positively associated with insulin sensitivity and the black ones were inversely associated. 3-OHB, 3-hydroxybutyrate; AC, Acylcanitine; Asn/Asx, Asparagine/aspartic acid; Glu/Glx, Glutamine/glutamic acid; Leu/Ile, Leucine/Isoleucine; NEFAs, Non-esterified fatty acids; OHpro, Hydroxyprolines.

APPENDIX

HAPO Study Cooperative Research Group

Field Centers: (North American Region) M Contreras, DA Sacks, W Watson (deceased) (Kaiser Foundation Hospital, Bellflower, California); SL Dooley, M Foderaro, C Niznik (Prentice Women's Hospital of Northwestern Memorial Hospital/Northwestern University Feinberg School of Medicine, Chicago, Illinois); J Bjaloncik, PM Catalano, L Dierker, S Fox, L Gullion, C Johnson, CA Lindsay, H Makovos, F Saker (MetroHealth Medical Center/Case Western Reserve University, Cleveland, Ohio); MW Carpenter, J Hunt, MH Somers (Women and Infants' Hospital of Rhode Island/Brown University Medical School, Providence, Rhode Island); KS Amankwah, PC Chan, B Gherson, E Herer, B Kapur, A Kenshole, G Lawrence, K Matheson, L Mayes, K McLean, H Owen (Sunnybrook and Women's College Health Sciences Center/University of Toronto, Toronto, Ontario); **(European Region)** C Cave, G Fenty, E Gibson, A Hennis, G McIntyre, YE Rotchell, C Spooner, HAR Thomas (Queen Elizabeth Hospital/School of Clinical Medicine and Research, University of the West Indies, Barbados); J Gluck, DR Hadden, H Halliday, J Irwin, O Kearney, J McAnee, DR McCance, M Mousavi, AI Traub (Royal Jubilee Maternity Hospital, Belfast, Northern Ireland); JK Cruickshank, N Derbyshire, J Dry, AC Holt, F Khan, C Lambert, M Maresh, F Prichard, C Townson (St. Mary's Hospital/Manchester University, Manchester, United Kingdom); TW van Haeften, AMR van de Hengel, GHA Visser, A Zwart (University Hospital/University Medical Center Utrecht, Utrecht, Netherlands); **(Middle Eastern/Asian Region)** U Chaovarindr, U Chotigeat, C Deerochanawong, I Panyasiri, P Sanguanpong (Rajavithi Hospital, Bangkok, Thailand); D Amichay, A Golan, K Marks, M Mazor, J Ronen, A Wiznitzer (Soroka Medical Center/Ben-

Gurion University, Beersheba, Israel); R Chen, D Harel, N Hoter, N Melamed, J Pardo, M Witshner, Y Yogev (Helen Schneider Hospital for Women, Rabin Medical Center/Sackler Faculty of Medicine, Tel-Aviv University, Petah-Tiqva, Israel); **(Austral-Asian Region)** F Bowling, D Cowley, P Devenish-Meares, HG Liley, A McArdle, HD McIntyre, B Morrison, A Peacock, A Tremellen, D Tudehope (Mater Misericordiae Mothers' Hospital/University of Queensland, Brisbane, Australia); KY Chan, NY Chan, LW Ip, SL Kong, YL Lee, CY Li, KF Ng, PC Ng, MS Rogers, KW Wong (Prince of Wales Hospital/Chinese University of Hong Kong, Hong Kong); M Edgar, W Giles, A Gill, R Glover, J Lowe, F Mackenzie, K Siech, J Verma, A Wright (John Hunter Hospital, Newcastle, Australia); YH Cao, JJ Chee, A Koh, E Tan, VJ Rajadurai, HY Wee, GSH Yeo (KK Women's and Children's Hospital, Singapore).

Regional Centers: D Coustan, B Haydon (Providence); A Alexander, DR Hadden (Belfast); O Attias-Raved, M Hod (Petah-Tiqva), JJN Oats, AF Parry (Brisbane)

Clinical Coordinating Center: A Collard, AS Frank, LP Lowe, BE Metzger, A Thomas (Northwestern University Feinberg School of Medicine, Chicago)

Data Coordinating Center: T Case, P Cholod, AR Dyer, L Engelman, M Xiao, L Yang (Northwestern University Feinberg School of Medicine, Chicago)

Central Laboratory: CI Burgess, TRJ Lappin, GS Nesbitt, B Sheridan, M Smye, ER Trimble (Queen's University Belfast, Belfast)

Steering Committee: D Coustan (Providence), AR Dyer (Chicago), DR Hadden (Belfast), M Hod (Petah-Tiqva), BE Metzger (Chicago), LP Lowe, ex officio (Chicago), JJN Oats (Brisbane), B Persson (Stockholm), ER Trimble (Belfast)

Data Monitoring Committee: GR Cutter, SG Gabbe, JW Hare, LE Wagenknecht

Consultants: Y Chen, J Claman, J King
